# Characterization and Differentiation of Petroleum-Derived Products by E-Nose Fingerprints

**DOI:** 10.3390/s17112544

**Published:** 2017-11-05

**Authors:** Marta Ferreiro-González, Gerardo F. Barbero, Miguel Palma, Jesús Ayuso, José A. Álvarez, Carmelo G. Barroso

**Affiliations:** 1 Department of Analytical Chemistry, Faculty of Sciences, IVAGRO, University of Cadiz, 11510 Puerto Real, Cádiz, Spain; marta.ferreiro@uca.es (M.F.-G.); gerardo.fernandez@uca.es (G.F.B.); carmelo.garcia@uca.es (C.G.B.); 2 Department of Physical Chemistry, Faculty of Sciences, University of Cadiz, 11510 Puerto Real, Cádiz, Spain; jesus.ayuso@uca.es (J.A.); joseangel.alvarez@uca.es (J.A.Á.)

**Keywords:** petroleum-derived products, characterization, fingerprints, E-Nose, gasoline, diesel

## Abstract

Characterization of petroleum-derived products is an area of continuing importance in environmental science, mainly related to fuel spills. In this study, a non-separative analytical method based on E-Nose (Electronic Nose) is presented as a rapid alternative for the characterization of several different petroleum-derived products including gasoline, diesel, aromatic solvents, and ethanol samples, which were poured onto different surfaces (wood, cork, and cotton). The working conditions about the headspace generation were 145 °C and 10 min. Mass spectroscopic data (45–200 *m*/*z*) combined with chemometric tools such as hierarchical cluster analysis (HCA), later principal component analysis (PCA), and finally linear discriminant analysis (LDA) allowed for a full discrimination of the samples. A characteristic fingerprint for each product can be used for discrimination or identification. The E-Nose can be considered as a green technique, and it is rapid and easy to use in routine analysis, thus providing a good alternative to currently used methods.

## 1. Introduction

Characterization of petroleum-derived products (PDPs) is an area of continuing importance in environmental science, for example for the identification of fuel spills [1]. Oil and fuel spills (accidental or illegal intentional operational discharges) are commonplace, but a full determination of the source is not always straightforward [2,3,4,5]. It is important to discriminate the type of spill to identify the source, evaluate the level of hazard and to employ the appropriate cleanup treatment [6,7]. Furthermore, very rapid identification is advantageous in that the appropriate clean-up procedure can be started as soon as possible. In most cases, the PDP is not found in isolation but adheres to different surfaces (wood, rocks, cloth, flooring etc.). The absorption characteristics of the substrates may affect the evaporation rate of the PDP and consequently the analysis and the identification [8].

GC-MS-based methods, including some sample preparation steps, are usually applied to characterize fuel-related products [9]. Using GC-MS, both chromatographic information and spectroscopic information can be used to characterize the samples. In any case, both chromatograms and spectra must be treated using the chemometric approach, in some cases using the total ion chromatogram (TIC), in other cases using extracted ion chromatograms [10], in combination with target compound analysis [11,12,13]. When the liquids are weathered, this method can be time-consuming and potentially subjective since the interpretation of the results is variable based on the analyst’s experience. Furthermore, this approach does not allow for full automation. Additionally, there is some information within the analytical results that is not used. One option to overcome the problems associated with the interpretation of GC-MS data is the application of chemometric tools. Chemometrics allows for the classification of data, the extraction of useful information, including discrimination between different groups of samples, in an almost automatic procedure [10,14]. Such procedures have included covariance mapping [15,16], principal components analysis (PCA) [5,11,17,18,19], linear discriminant analysis (LDA) [19], quadratic discriminant analysis (QDA) [20], artificial neural networks [5], soft independent modeling of class analogy (SIMCA) [21], cluster analysis [22], self-organizing feature maps [21], and fuzzy rule-building expert system classification [23].

In some cases, the quantification of compounds in the chromatogram is not needed as the chromatographic pattern can be used to characterize samples. This procedure is very simple and also very useful, as it can produce fingerprints that enable very fast sample identification. However, this procedure cannot be applied in different laboratories/equipment from those used for method development because small differences in the retention times would produce unreliable results. One strategy to overcome the challenges related to changing retention time is related to the analysis of the total ion spectrum (TIS), which is obtained by summing the intensities of each *m*/*z* signal over all chromatographic results. The TIS is time-independent and has been used as a new method for the rapid classification of petroleum-derived products after several reference samples have been included in a database [24], and this method has already been applied.

GC-MS has been demonstrated to be helpful in this field, but relatively long analysis times are needed. Therefore, the development of methods that do not require chromatographic separation for the solution of several analytical problems is currently of interest in many fields. A fingerprint of the sample obtained by integrating all the components is sufficient in some cases [25].

The application of different spectroscopic techniques—such as NIR, FT-IR, or Raman—combined with chemometric tools has also been described in the literature as an alternative to chromatographic techniques [26,27,28]. These spectroscopic techniques have several advantages since they are non-destructive, are easy to use, are cheap, can be applied in situ, and require limited or no sample preparation. However, these methods also have certain drawbacks, as they provide limited information about specific components and they do not have as high a sensitivity that the mass detector has.

In the study described here, we proposed a non-separative analytical method based on E-Nose using a mass spectrometer as detection system. This system can be considered an E-Nose because it detects volatile compounds, records data and uses data treatment to develop models and fingerprints [29]. In recent years, E-Nose has been heavily developed for several applications, and some portable systems have even been used for the classification of gases [30,31].

This system has been optimized in a previous study, and the resulting method validated, for analysis of gasoline samples with different research octane numbers (RONs) [32,33]. In the response pattern obtained by E-Nose, the final mass spectrum is equivalent to the summed ion spectra and this is obtained in only a few minutes because there is no chromatographic separation. The resulting total ion spectrum is characteristic of the sample being analyzed and so can be utilized as a fingerprint of each sample [34].

This technique has been previously applied to studies related to PDPs. In this sense, Feldhoff et al. presented a study in which they compared the performance of a different system for the characterization of diesels and they have concluded that MS data were obtained easily and were more reproducible [35]. In the same way, Pavón et al. used the headspace mass spectrometer to analyze methyl tert-butyl ether (MTBE) in gasoline samples. In all cases, the chemometric treatment of the spectroscopic results is essential in order to extract the information contained within the signal profile [36].

The aim of the work described here was to develop a new method to determine the presence/absence of petroleum-derived products using fingerprints. Such an approach would enable the fast and easy identification of different PDPs (gasoline, diesel, ethanol, and aromatic solvents) adhered to different surfaces (wood, cork, and cotton sheet) using an E-Nose technique combined with chemometric tools.

## 2. Experimental Section

### 2.1. Samples

A total of 39 samples were prepared using different petroleum-derived products: 3 gasoline samples with 95 research octane number purchased from 3 different local petrol stations and different brands, G1 (Repsol), G2 (Cepsa), and G3 (BP); 3 diesel samples, also obtained from 3 different local petrol stations and brands, D1 (Cepsa), D2 (Shell), and D3 (Repsol); 3 commercial ethanol samples obtained from different commercial brands in local stores, E1 (ethanol 96%, Alcoholes Monplet, S.A., Barcelona, Spain), E2 (ethanol absolute, Merck KGaA, Darmstadt, Germany), and E3 (ethanol 96%, Panreac Química, S.A.U., Barcelona, Spain); and 3 different aromatic organic solvents common in regular laboratories, A1 (xylene), A2 (toluene), and A3 (bromobenzene), all of which were purchased from Panreac Química, S.A.U. Liquids were added to three types of substrate: pine wood (pw), natural cork (co), and 100% cotton sheet (sh). Substrates without any petroleum-derived products were also analyzed. Samples are denoted by the liquid code followed by the substrate code, i.e., G1co for cork with Gasoline 1, D2sh for sheet with Diesel 2, and so on. Substrates without liquids are referred to as N (none), followed by the substrate code: Npw, Nco, and Nsh.

Each type of liquid (80 μL) was added to the pieces of different materials (5 cm × 5 cm) inside the E-Nose glass vials, and they were analyzed in these vials. All samples were prepared in duplicate, and the average mass spectrum for each case was used for the study.

### 2.2. Acquisition of E-Nose Spectra

The substrate samples were analyzed on an Alpha Moss (Toulouse, France) E-Nose system composed of an HS 100 static headspace autosampler and a Kronos quadrupole mass spectrometer (MS). The samples were contained in 10 mL sealed vials (Agilent Crosslab), and these were then placed in the autosampler oven to be heated and agitated in order to generate the headspace. Headspace was finally taken from the vial using a gas syringe and injected into the mass spectrometer. The gas syringe was heated above the sample temperature (+5 °C) to avoid condensation phenomena. Between each sample injection, the gas syringe was flushed with carrier gas (nitrogen) to avoid cross-contamination.

The experimental conditions for the headspace sampler were as follows: incubation temperature: 145 °C; incubation time: 10 min; agitation speed: 500 rpm; syringe type: 5 mL; syringe temperature: 150 °C; flushing time: 120 s; fill speed: 100 μL/s; injection volume: 4.5 mL; injection speed: 75 μL/s. The total time per sample was approximately 12 min, i.e., approximately 10 times faster than regular methods using sample preparation plus GC-MS analysis.

The components in the headspace of the vials were injected directly into the mass detector without any chromatographic separation or sample pre-treatment. On using this process, for any given measurement, the resulting mass spectrum gave a typical signal of the product. Ion electron impact spectra were recorded in the range *m*/*z* 45–200. Instrument control was achieved using the residual gas analysis and the Alpha Soft 7.01 software package.

### 2.3. Data Analysis and Software

The mass spectra were normalized to the maximum intensity for each sample. Multivariate analysis of the data, which included hierarchical cluster analysis (HCA), PCA, and LDA, was performed using the statistical computer package SPSS 22.0 (SPSS Inc., Chicago, IL, USA).

## 3. Results and Discussion

In a previous study, an E-Nose-based method was optimized for the analysis of gasoline samples with different research octane numbers [29]. The optimized HS conditions for the discrimination of the different gasoline samples were as follows: a 145 °C incubation temperature, a 10 min incubation time, and an 80 μL sample volume.

The present work is focused on the applicability of this optimized method for the discrimination of different petroleum-derived products (PDPs) supported on different solid materials. In this case, 80 μL of each PDP was added to different substrates and these samples were analyzed by the E-Nose using the optimized method for HS generation. The PDP was placed on the surface of the material in order to evaluate whether the E-Nose is able to detect the PDP even when it is adsorbed on a surface, and to ascertain if signals from the PDP can be differentiated from the signals due to the supporting material.

**Figure 1:**
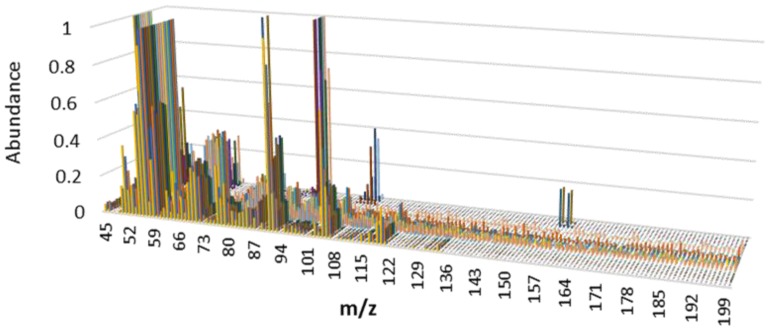
Mass spectra for all samples, including supported petroleum-derived product (PDP) samples and materials without PDPs (*n* = 39) analyzed by an electronic nose system.

All of the spectra were recorded in the range *m*/*z* 45–200, and the resulting spectra were normalized to a total intensity of one. The spectra obtained for all the liquids upon heating the samples for 10 min at 145 °C are shown in Figure 1.

Visual inspection of the spectra (Figure 1) shows differences between some of the PDP samples. For instance, ethanol samples are the only ones that present signal at *m*/*z* 45, and one of the aromatic solvents is the only liquid that presents signals at *m*/*z* 156 and *m*/*z* 158. However, even gasoline and diesel samples have several common signals in the E-Nose system. It can be seen that drawing any conclusion on the presence/absence of a PDP based on the visual pattern recognition of the total mass spectrum is time-consuming. Besides, this procedure is highly dependent on the skill and experience of the analyst and does not allow automation. Therefore, the possibility of developing fingerprints that would enable automatic data interpretation for fast PDP identification is of great interest in this field. In order to develop the PDP fingerprints, it is necessary to identify the signals in the spectra that allow for the discrimination of different liquids. It is therefore necessary to apply chemometric tools in order to extract the information contained in the signal profiles (i.e., to identify the appropriate *m*/*z* signals) that create a characteristic pattern or fingerprint for each type of sample.

**Figure 2:**
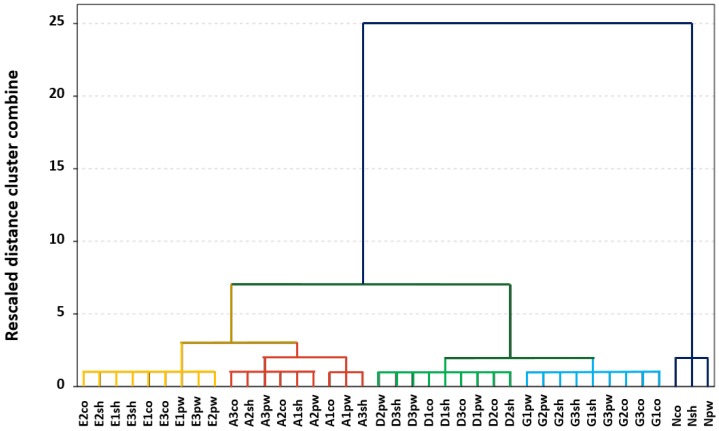
Dendrogram obtained from the hierarchical cluster analysis using the Ward method.

This work is the first time that the E-Nose has been used with this aim, and it was therefore necessary to check the applicability of the system in the characterization of different PDPs (gasoline, diesel, ethanol, aromatic solvents) adsorbed on different surfaces. For this reason, an exploratory tool—namely HCA—was applied to all of the *m*/*z* values (45–200 *m*/*z*) as variables to form groups. A total of 39 samples (9 substrates containing gasoline, 9 diesel, 9 ethanol, 9 aromatic solvents, and the 3 materials without any liquid) were analyzed, two samples were analyzed for each PDP, and the average spectrum was employed in each case. The results of the HCA are represented in the dendrogram in Figure 2. Clear differentiation of some groups for the samples can be clearly observed.

A primary trend for grouping can be seen in Figure 2 according to the presence or absence of liquid in the substrates, since all of the substrates that were free of PDPs (Nco, Nsh, Npw) are separated from the rest of the samples in a different cluster, namely Cluster 2 (dark blue). Based on these results, E-Nose allows the detection of the studied PDPs in any material on which they are supported. Cluster 1 contains all of the substrates with the different PDPs, and all of the samples containing the same type of liquid were grouped together in different sub-clusters: samples with ethanol in Cluster 1.1, those with aromatic solvents in Cluster 1.2, those with diesel in Cluster 1.3, and those with gasoline in Cluster 1.4. From the HCA it can also be observed that samples containing diesel and gasoline are grouped closely, and a similar trend is observed with samples containing ethanol and aromatic solvents.

This tendency to cluster according not only to the presence/absence of the liquids but also to the type of PDP indicates that the data from the E-Nose analyses used to perform the HCA are related to the compounds that are responsible for the characterization of different liquids, also supporting materials without PDP.

**Figure 3:**
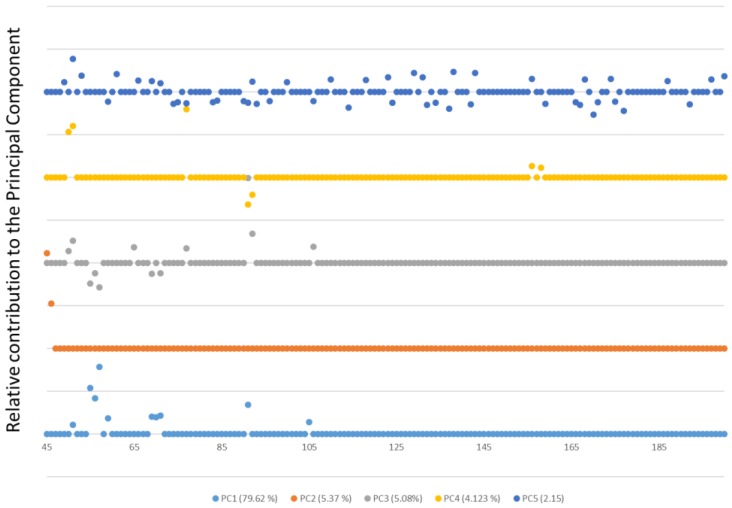
Loadings for the first five factors resulting from principal components analysis (PCA) using the signals from the E-Nose system.

**Figure 4:**
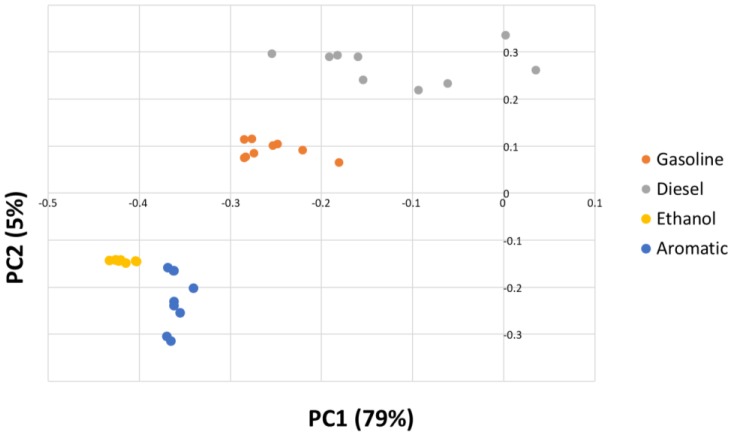
PC1–PC2 Score plot for all the samples containing PDP based on the E-Nose data.

Based on this result, a principal component analysis (PCA) was performed on the data set. PCA will provide information about specific *m*/*z* signals related to the maximum variability between samples. Additionally, the contributions by the *m*/*z* to different factors will help in the construction of the discrimination model. According to this PCA, seven factors were required to explain at least 99% of the information contained in the body of mass spectra for the samples. After analyzing the loadings for the resulting factors, it was seen that Factor 1 showed a clear contribution from specific *m*/*z* signals. The loadings for the first five factors are depicted in Figure 3. It can be seen that the first factor explains 79.62% of the variance and its signals (*m*/*z*), except for the signal at *m*/*z* 105, are below *m*/*z* 100. Factor 2 accounts for 5.37% of the information and it only shows a signal at *m*/*z* of 45. Factor 3 accounts for 5.08% of the information and, as in the case of Factor 1, all of the signals except for *m*/*z* 106 are below *m*/*z* 100. Factor 4 and Factor 5 account for less than 5% of the variance. A biplot for scores on the PC2–PC1 plane for all the samples with PDP is shown in Figure 4. It can be observed that four different groups of samples (which correspond to the four types of PDP) are clearly distinguished using only the first two factors (PC1&PC2). Besides, it can also be seen how both factors are needed for a full separation of the samples regarding the type of PDP added to the substrate.

Based on the tendency to grouping shown in HCA and PCA, and since the objective of this study was to develop a methodology for the characterization of the samples, a supervised technique, namely LDA, was applied to the whole body of mass spectra (*m*/*z* 45–200). In an effort to achieve a more robust discrimination prior to running the LDA, around 70% (*n* = 28) of the samples were randomly selected as a training set to obtain the discriminant functions and the remaining 30% (*n* = 11) of the samples were used as a validation set. In order to identify whether there are specific *m*/*z* values in the mass spectra that are more significant than others when classifying the PDPs, and with the aim of developing a fingerprint for each PDP that allows for automatic classification of the samples, a stepwise discriminant analysis was performed. Five groups were defined, specifically samples with gasoline, diesel, ethanol, or aromatic liquids and samples without PDPs.

The resulting linear discriminant functions enabled full discrimination between the five groups of PDP samples. Samples from both sets (calibration and validation set) were unambiguously assigned to their corresponding group. The *m*/*z* values selected to develop the discrimination function were as follows: *m*/*z* 45, *m*/*z* 47, *m*/*z* 56, *m*/*z* 57, *m*/*z* 59, *m*/*z* 64, *m*/*z* 65, *m*/*z* 70, *m*/*z* 71, *m*/*z* 85, *m*/*z* 92, *m*/*z* 120, *m*/*z* 126, *m*/*z* 138 and *m*/*z* 145, and *m*/*z* 193. This means that there are different spectroscopic areas in the *m*/*z* studied that are related to the different PDPs and these are required to obtain a full discrimination of the samples.

**Figure 5:**
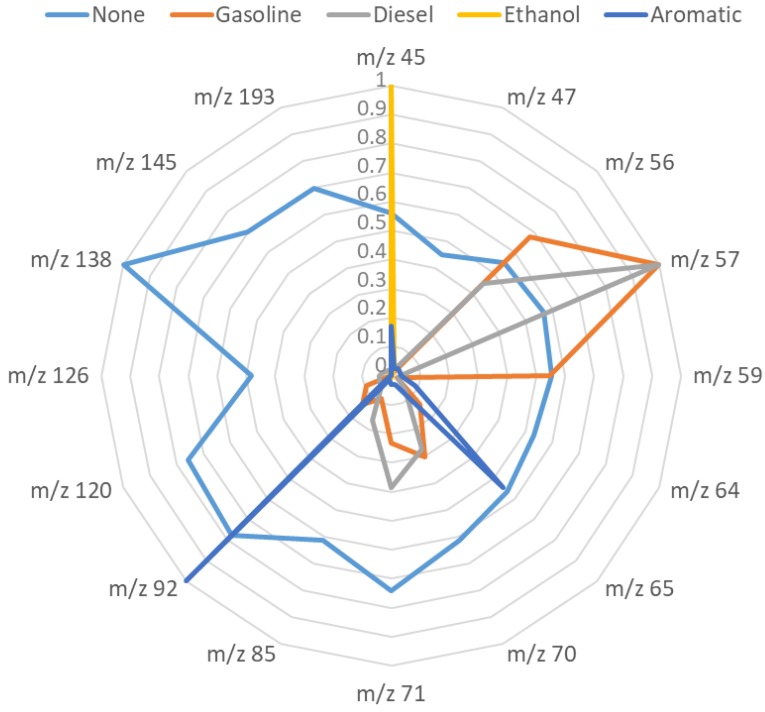
PDP fingerprints obtained by displaying the values of the *m*/*z* selected in the linear discriminant analysis (LDA).

When only the abundance values of these *m*/*z* are represented, the different fingerprint obtained for each type of PDP can be observed (Figure 5). All of the values were normalized to the base peak at 100%. A limited number of *m*/*z* values are above 50% of the maximum intensity for each fingerprint. The scenario is completely different for substrates without PDPs (none). For these samples, most of the *m*/*z* values of its fingerprint are above 0.5 (50% of the maximum intensity).

In contrast, samples that contain PDPs show very few important signals (*m*/*z* values above 0.5) and, in the case of substrates with ethanol, not even a single signal of this magnitude.

In samples with ethanol, the characteristic signal is *m*/*z* 45, in samples with aromatic solvents there are two such signals, *m*/*z* 92 and *m*/*z* 65, and *m*/*z* also seems to be important even though the intensity of *m*/*z* 45 is below 0.5. In samples with gasoline or diesel, the maximum signal is *m*/*z* 57, but the other signals are not the same or they do not have the same intensity. Gasoline samples show high intensity signals at *m*/*z* 59, but this signal is virtually absent from samples with diesel. The same behavior can be seen for *m*/*z* 56 and *m*/*z* 59, which are also intense in gasoline samples, whereas in diesel samples *m*/*z* 56 is below 0.5 and *m*/*z* 59 is almost absent. In addition, the intensities and the ratios of the rest of the signals are also different for each PDP, thus giving different fingerprints that can be used to discriminate the different PDPs. This finding is illustrated in Figure 5 for samples with gasoline and diesel. Both types of sample present signals at *m*/*z* 70 and *m*/*z* 71 but not above 0.5. However, the ratio between these *m*/*z* (70/71) is above 1 in the case of gasoline samples but below 1 in the case of diesel samples. As a consequence, these signals are also useful for the characterization although they are not particularly intense.

## 4. Conclusions

Based on the results described above, it can be concluded that the E-Nose system is able to identify not only the presence/absence of the studied PDPs but also the type of PDP adhered to different surfaces. Therefore, if a database containing PDP fingerprints is developed in a real situation, then the identification of PDPs by matching unknown fingerprints with those from the database or with a suspect sample can be easily achieved.
